# Comparative genomics analysis of six Cutibacterium acnes strains isolated from contaminated platelet concentrates

**DOI:** 10.1099/acmi.0.000938.v3

**Published:** 2025-04-08

**Authors:** Annika Flint, Dilini Kumaran, Kelly Weedmark, Franco Pagotto, Sandra Ramirez-Arcos

**Affiliations:** 1 Microbiology Research Division, Bureau of Microbial Hazards, Food and Nutrition Directorate, Health Canada, Ottawa, Ontario, Canada; 2 Donation Policy & Studies, Canadian Blood Services, Ottawa, Ontario, Canada; 3 Department of Biochemistry, Microbiology and Immunology, University of Ottawa, Ottawa, Ontario, Canada; 4 Listeriosis Reference Centre, Microbiology Research Division, Bureau of Microbial Hazards, Food and Nutrition Directorate, Health Canada, Ottawa, Ontario, Canada

**Keywords:** *Cutibacterium acnes*, genome sequencing, platelet concentrates

## Abstract

*Cutibacterium acnes* is a bacterial skin commensal that is often isolated during routine testing of blood products like platelet concentrates (PCs). Due to the slow-growing nature of this bacterium in culture media, *C. acnes* contaminated PCs are often transfused into vulnerable patients before retrieval of these units can be initiated. This study aimed at obtaining the whole-genome sequence of six *C. acnes* isolates derived from contaminated PCs, comparing and assessing their genetic backgrounds. Furthermore, the whole genomes of the PC isolates were compared to clinical isolates obtained from different sites and types of infection. The results indicate that these PC isolates assessed belong to four phylotypes, namely IA, IB, II and III. Whole-genome comparisons identified differences in the virulence profiles of the isolates and provide a foundation for future studies aimed at evaluating the risk to transfusion patients by determining whether the expression of virulence factors is impacted in the PC storage environment.

**Impact Statement**

Canadian Blood Services screens platelet concentrates (PCs) for bacterial contamination with the automated culture BACT/ALERT system. *Cutibacterium acnes*, a skin flora anaerobic aerotolerant bacterium, is the predominant PC contaminant: ~40% of PC units issued to hospitals are contaminated with *C. acnes* and likely transfused prior to detection due to the slow-growing nature of this bacterium in the BACT/ALERT culture system. While this organism does not reach clinically significant levels during PC storage to cause acute septic transfusion reactions, *C. acnes* may pose an enhanced risk of chronic infection in transfusion patients. The impact of the PC storage environment on other anaerobic bacteria remains to be studied.

## Data Summary

Genome sequences and reads are available in GenBank and the Sequence Read Archive under the accession numbers listed in Table 1 (NCBI Bioproject PRJNA907754).

**Table 1.** Assembly metrics of closed genomes and NCBI accession numbers for *C. acnes* isolates (NCBI Bioproject PRJNA907754)

Metric	CBS-BPNBT19269	CBS-BPNBT19195	CBS-BPNBT19329	CBS-BPNBT19223	CBS-BPNBT19227	CBS-BPNBT19153
Chromosome (bp)	2,547,251	2,560,088	2,492,467	2,578,943	2,594,562	2,550,536
G+C content (mol%)	60.09	60.02	60.04	59.94	59.94	60.04
Annotated features	2,504	2,473	2,401	2,480	2,489	2,486
Genes	2,369	2,313	2,194	2,145	2,162	2,339
rRNAs	9	9	9	9	9	9
tRNAs	45	45	45	45	45	46
ncRNAs	4	4	3	4	4	4
Pseudogenes	77	102	150	277	278	88
CRISPRs	0	0	1	0	0	0
Phylotype	IA	IB	II	III	III	IA
GenBank accession no.	CP114012.1	CP114011.1	CP114010.1	CP114009.1	CP114008.1	CP114007.1
Illumina accession no.	SRR22502664	SRR22502664	SRR22502662	SRR22502660	SRR22502658	SRR22502666
Nanopore SRA accession no.	SRR22502663	SRR22502663	SRR22502661	SRR22502659	SRR22502657	SRR22502665
City, Province andCountry of origin	Edmonton, Alberta, Canada	British Columbia and Yukon,Canada	Edmonton, Alberta,Canada	Calgary,Alberta,Canada	Brampton, Ontario,Canada	Ottawa,Ontario,Canada
Year	2019	2019	2019	2019	2019	2019

## Introduction

Platelet concentrates (PCs) are therapeutic blood products manufactured at Canadian Blood Services to treat patients with platelet dysfunction or to staunch blood loss due to trauma [1,2]. Though rare, bacterially contaminated PCs have been involved in serious transfusion reactions caused by the proliferation of contaminating bacteria during PC storage (20–24 °C, gas permeable bags and constant agitation) [3,4]. The donor’s skin serves as a source of bacterial contamination and is introduced into donated blood during venipuncture [5]. PC screening for bacterial contamination at Canadian Blood Services involves sampling at≥36 h post-collection, inoculation of aerobic and anaerobic culture bottles and post-sampling quarantine for≥6 h before units are released into inventory with a negative-to-date testing result [6]. Cultures are incubated in the automated BACT/ALERT system for a maximum of 7 days. When a positive BACT/ALERT result is obtained, source PC units found in inventory are discarded; however, if units had been issued, the hospital blood bank is immediately informed, and patients who received contaminated units are managed by the attending physicians according to hospital protocols. Haemovigilance data obtained at Canadian Blood Services from 2017 to 2023 indicated that~72% of bacteria isolated from contaminated PCs were identified as the anaerobic (aerotolerant) member of the skin flora *Cutibacterium acnes*. Furthermore, ~42% of bacterially contaminated PC units were issued to hospitals and transfused before positive BACT/ALERT results. Most of those units (~98%) were contaminated with *C. acnes*, which grows slowly in BACT/ALERT anaerobic culture media. The transfusion of *C. acnes* from PCs does not result in severe transfusion reactions, as it is unable to proliferate in the aerobic PC storage environment [7] and consequently does not reach clinically significant bacterial loads (≥10^5^ c.f.u. ml^−1^) as observed with aerobic or facultative PC contaminants [8]. While the acute risks associated with the transfusion of *C. acnes* contaminated blood products are considered very low, the effects of delayed reactions in vulnerable patients have yet to be ascertained. Notably, in recent years, *C. acnes* has been implicated in several serious chronic infections involving prosthetic devices (infectious endocarditis, prosthetic joint infections) [9,10] and spinal and brain infections [11,12], and has even been linked to prostate cancer [13].

This report describes genomes of six *C. acnes* isolates identified in 2019 during routine bacterial PC screening performed at Canadian Blood Services. Analyses of these genomes and comparisons to clinical *C. acnes* provide a foundation for future studies aimed at determining the impact of the PC environment on the expression profile of *C. acnes* virulence factors.

## Methods

### Bacterial growth conditions

*C. acnes* was isolated from six positive BACT/ALERT culture bottles (CBS-BPNBT19269, CBS-BPNBT19195, CBS-BPNBT19329, CBS-BPNBT19223, CBS-BPNBT19227 and CBS-BPNBT19153) obtained during routine bacterial screening of PCs at Canadian Blood Services. BACT/ALERT positive results were obtained after the PC units had been transfused. However, no acute septic transfusion reactions were reported for PCs contaminated with any of the six *C. acnes* strains used in this study. Bacterial isolation and identification were carried out as per standard protocols established at Canadian Blood Services [6]. Briefly, samples from the culture bottles were subcultured onto anaerobic blood agar CDC (Fisher Scientific, ON, Canada) or OxyPras anaerobic agar media (Oxyrase, Ohio, USA) and incubated for up to 5 days at 37 °C under anaerobic conditions. Isolates were identified using the VITEK 2 system with the anaerobic (ANC) card following manufacturer recommendations (bioMérieux, QC, Canada).

### MIC assays

MIC assays against rifampicin, mupirocin and trimethoprim (E-test strips, bioMérieux, QC, Canada) and bacitracin (discs, Oxoid, Thermo Fisher, ON, Canada) were determined as per manufacturers’ instructions, with *C. acnes* ATCC 6919 and *Staphylococcus aureus* ATCC 29213 used as control strains.

### DNA extraction and sequencing

Bacterial colonies were grown for 72 h at 35^ °^C on Brucella agar (Oxyrase Inc.). The Zymo Quick-DNA high-molecular-weight MagBead kit (Zymo Research Corp.) was used to extract DNA from single colonies according to manufacturer recommendations (with optional RNAse A treatment). Paired-end Illumina sequencing was performed as per manufacturer protocols using the NexteraXT DNA Library Preparation Kit, Nextera DNA UD Indexes and MiSeq instrument (v3 chemistry, 2×300 bp) (Illumina Inc.). The rapid barcoding sequencing kit (SQK-RBK004) and 1D MinION chemistry (R9.4 FLO-MIN106 flow cell) were used for Oxford Nanopore sequencing as per manufacturer instructions (Oxford Nanopore Technologies).

### Bioinformatic analysis

#### Read processing, *de novo* genome assembly and annotation

Raw Illumina reads were processed using FastP (v0.20.1) [14]. Oxford Nanopore reads were processed using Guppy GPU v6.0.1+652ffd1 (base calling, demultiplexing and adapter trimming) and Filtlong v0.2.0 (github.com/rrwick/Filtlong) to remove reads<5 kb. *De novo* hybrid assemblies were generated with Unicycler (v 0.5.0) in normal mode [15]. Error corrections were performed with Medaka (v1.1.3, github.com/nanoporetech/medaka), Polypolish v0.4.3 [16] and Polca v4.0.5 (github.com/alekseyzimin/masurca). Assemblies were annotated and analysed with PGAP v 2022-10-03.build6384 (github.com/ncbi/pgap) and QUAST v5.0.2 (github.com/ablab/quast).

#### *In silico* phylotyping and genome-wide comparisons

*In silico* phylotyping of *C. acnes* strains was performed using 16S rRNA, *sodA*, *atpD*, *recA*, *ATPase* and *fic* family toxin genes as described by Barnard *et al.* [17]. Briefly, genes from our isolates and 35 complete *C. acnes* GenBank genomes were identified with blastn (v.2.12.0+) using -perc_identity 90 and -evalue 0.001 [18,19]. The concatenated genes were aligned with MUSCLE using MEGA (v11.0.13) [20]. Maximum likelihood phylogenetic trees (Tamura–Nei model) were constructed with MEGA using the aligned sequences and 1,000 bootstrap replicates. Genome-wide comparisons of *C. acnes* were performed using Minimap2 v2.24-r1122 with the -x asm5 parameter [21] and paftv v0.1.0 using the -X option (github.com/MoinSebi/paftv). AliTV v1.0.6 (identity 70% and length 10k) [22] and Adobe Illustrator CS5 were used to visualize allignments. Antimicrobial and stress resistance genes were identified using antimicrobial resistance (AMR) FinderPlus (AMRFinderPlus v3.11.14 [23]), with the --plus option, coverage and identity settings -c 90 and -i 0.3 and the AMRfinder database (v2023-04-17.1). Virulence genes were identified using the virulence factor database ([24]; http://www.mgc.ac.cn/VFs/, accessed 07-19-2023) and by manual inspection of Prokaryotic Genome Annotation Pipeline (PGAP) annotations. All computational analyses used default settings except where specified.

#### Whole-genome comparison of clinical and PC-derived *C. acnes* isolates

Core genomes of our isolates and 24 complete/draft *C. acnes* GenBank genomes were aligned using Parsnp (v.1.2 [25]; github.com/marbl/parsnp) with *C. acnes* CBS-BPNBT19269 as the reference strain. Maximum likelihood phylogenetic trees (Tamura–Nei model) were constructed with MEGA (v11.0.13) [19] using the aligned sequences and 1,000 bootstrap replicates.

## Results and discussion

### Whole-genome sequencing and phylotyping of *C. acnes* isolates from PCs

**Fig. 1.**
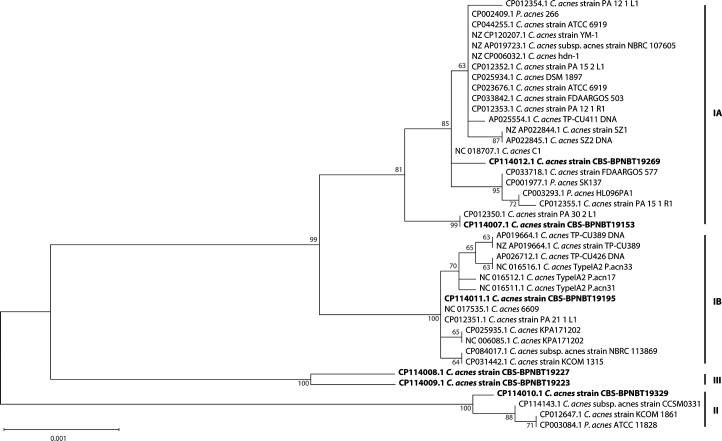
*In silico* phylotyping of *C. acnes* genomes. Multi-gene alignments were generated using MUSCLE and a maximum likelihood tree constructed using MEGA (v11.0.13) with 1,000 bootstrap replicates. The scale bar represents the phylogenetic distance expressed as nucleotide substitutions per site. Bootstrap values are shown at each node. Reference sequences were obtained from GenBank with tips labelled by accession number and strain name. The *C. acnes* strains from this study are in bold and phylotypes for each strain are shown on the right.

Metrics for the six *C. acnes* PC genomes are shown in Table 1. The closed genomes range in size from 2.4 to 2.6 Mb, G+C content from 59.94– to 60.09 mol% and the number of genes from 2,145 to 2,369. Notably, none of these genomes contain plasmids, which have been shown to elicit inflammatory responses and confer antimicrobial resistance in *C. acnes* [26,27]. *In silico* phylotyping (Fig. 1) was consistent with previous PCR results [28], indicating that isolates CBS-BPNBT19269 and CBS-BPNBT19153 are phylotype IA, CBS-BPNBT19195 and CBS-BPNBT19329 are phylotypes IB and II, respectively, and CBS-BPNBT19223 and CBS-BPNBT19227 belong to III. Correlation between *C. acnes* phylotype and invasiveness has been shown for phylotypes IA, IB and II; however, specific genetic contributing factors have not been identified [29]. Several reports have identified putative virulence genes in *C. acnes* [30]; however, very little is known about the mechanisms involved in *C. acnes* pathogenesis. Notably, within the context of the transfusion setting, we have shown that the PC storage environment elicits the upregulation of virulence factors in other transfusion relevant bacteria such as *S. aureus* and *Staphylococcus epidermidis* [31,32]. Therefore, the putative virulence genes identified in *C. acnes* PC isolates serve as worthy candidates for the evaluation of the modulation of *C. acnes* virulence in the PC storage milieu.

### Virulence determinants and antimicrobial resistance

#### Virulence determinants

**Fig. 2.**
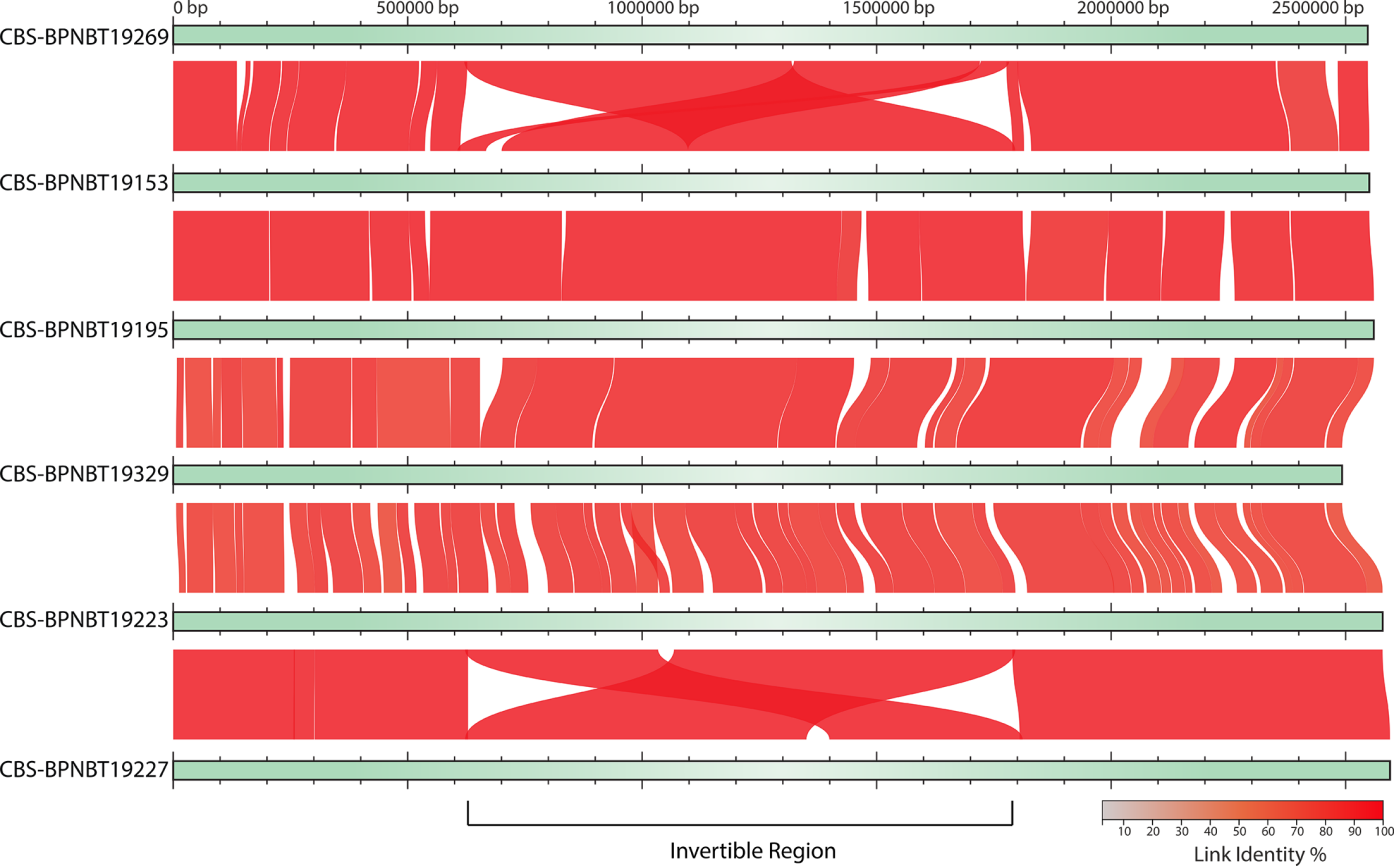
Comparative genomic analysis of *C. acnes* strains shows an invertible region. Multiple genome alignment of *C. acnes* strains. Alignments were generated using MiniMap2 and visualized using AliTV. Genome positions are shown along the top of the alignments (bp). Linked segments represent orthologous gene pairs between genomes and breaks in synteny are shown as blank spaces. Colour denotes the per cent similarity of links between isolates.

Genome-wide comparisons between the *C. acnes* isolates show a large (~1.2 Mb) chromosomal invertible region (Fig. 2) flanked by ribosomal operons, which has been reported previously in *C. acnes* and *Pseudomonas aeruginosa* [33,34]. Though the exact impact these invertible regions have on *C. acnes* virulence is unknown, the presence of these regions in cystic fibrosis-derived *P. aeruginosa* corresponded to poorer clinical outcomes and was suggested to play a role in global transcriptional changes impacting virulence [34].

Putative virulence genes were identified and categorized for *C. acnes* genomes (Table 2). All isolates contain genes associated with adherence, biofilm formation, secretion systems, immune evasion, tissue invasion and degradation. Though the role of many of these genes is not well defined in *C. acnes* in the context of its pathogenicity, they are important virulence factors in pathogenic bacteria. For instance, sortase and GroEL play a role in anchoring surface proteins to the bacterial cell wall and protein folding, respectively [35,36]. Similarly, the ability to produce a capsule and biofilms has been reported to play an important role in evading the immune response and establishing persistent chronic infections in the clinical setting [37,38]. This suggests that though *C. acnes* is often dismissed as a cause for concern in clinical and transfusion settings, this bacterium harbours an arsenal of elements which could lead to opportunistic infections causing significant morbidity, as has been reported in recent years [30].

**Table 2.** Virulence genes identified in *C. acnes* genomes using the virulence factor database and PGAP

Class	Virulence factor	Related gene	Gene encoded					
			BPNBT-19269	BPNBT-19195	BPNBT-19329	BPNBT-19223	BPNBT-19227	BPNBT-19153
**Adherence**	GroEL	*groEL*	✓	✓	✓	✓	✓	✓
	Sortase F	*srtF*	✓	✓	✓	✓	✓	✓
**Iron uptake**	ABC-type haeme transporter	*hmuT*	✓	✓	✓	✓	✓	✓
		*hmuU*	✓	✓	✓	✓	✓	✓
	Siderophore-dependent iron uptake system	*irp6A*	✓	✓	✓	✓	✓	✓
		*irp6B*	✓	✓	✓	✓	ND	ND
		*irp6C*	✓	✓	✓	✓	✓	✓
**Regulation**	Diphtheria toxin repressor DtxR	*dtxR*	✓	✓	✓	✓	✓	✓
	(p)ppGpp synthesis and hydrolysis	*relA*	✓	✓	✓	✓	✓	✓
	MprA/B	*mprA*	✓	✓	✓	✓	✓	✓
	PhoP/R	*phoP*	✓	✓	✓	✓	✓	✓
	RegX3	*regX3*	✓	✓	✓	✓	✓	✓
	Sigma A	*sigA/rpoV*	✓	✓	✓	✓	✓	✓
	Sigma H	*sigH*	✓	✓	✓	✓	✓	✓
**Amino acid and purine metabolism**	Glutamine synthesis	*glnA1*	✓	✓	✓	✓	✓	✓
	Lysine synthesis	*lysA*	✓	✓	✓	nd	nd	✓
**Anaerobic respiration**	Nitrate reductase	*narG*	✓	✓	✓	✓	✓	✓
		*narH*	nd	nd	nd	✓	✓	nd
		*narI*	nd	nd	nd	✓	✓	nd
**Anti-apoptosis factor**	NuoG	*nuoG*	✓	✓	✓	✓	✓	✓
**Antiphagocytosis**	Capsule	*gnd*	✓	✓	✓	✓	✓	✓
		*ugd*	✓	✓	✓	✓	✓	✓
**Immune evasion**	LPS	*acpXL*	✓	✓	✓	✓	✓	✓
**Phagosome arresting**	Nucleoside diphosphate kinase	*ndk*	✓	✓	✓	✓	✓	✓
**Stress adaptation**	Iron-co-factored SOD	*sodA*	✓	✓	✓	✓	✓	✓
**Exoenzyme**	Lipoyl synthase	*lipA*	✓	✓	✓	✓	✓	✓
	Lipoyl (octanoyl) transferase	*lipB*	✓	✓	✓	✓	✓	✓
	CAMP factor	*cfb (CAMP1)*	✓	✓	✓	✓	✓	✓
		*cfb (CAMP2)*	✓	✓	✓	✓	✓	✓
		*cfb (CAMP3)*	✓	✓	✓	✓	✓	✓
		*cfb (CAMP4)*	✓	✓	✓	✓	✓✓	✓
		*cfb (CAMP5)*	✓	✓	✓	✓	✓	✓
	MarP family serine protease	*marP*	✓	✓	✓	✓	✓	✓
**Secretion system**	Preprotein translocase subunit	*secG*	✓	✓	✓	✓	✓	✓
	Protein translocase subunit	*secD*	✓	✓	✓	✓	✓	✓
	Protein translocase subunit	*secF*	✓	✓	✓	✓	✓	✓
	Preprotein translocase subunit SecA	*secA*	✓	✓	✓	✓	✓	✓
	Preprotein translocase subunit	*secY*	✓	✓	✓	✓	✓	✓
	Preprotein translocase subunit	*secE*	✓	✓	✓	✓	✓	✓
	Preprotein translocase subunit	*yajC*	✓	✓	✓	✓	✓	✓
	Membrane protein insertase	*yidC*	✓	✓	✓	✓	✓	✓
	Signal recognition particle-docking protein	*ftsY*	✓	✓	✓	✓	✓	✓
	Signal recognition particle protein	*Ffh*	✓	✓	✓	✓	✓	✓
**Biofilm and quorum sensing**	S-ribosylhomocysteine lyase	*luxS*	✓	✓	✓	✓	✓	✓
	Acetyl-coenzyme A synthase	*acsA*	nd	nd	nd	nd	nd	✓
**Host-tissue degradation and inflammation**	Lysophospholipase	*ytpA*	✓	✓	✓	✓	✓	✓

✓ -✓-– indicates the presence of the indicated gene,; ✓✓-– indicates the presence of two copies of the indicated gene; nd: gene not detected.

Furthermore, a cluster of genes involved in carbohydrate uptake (ABC transport permeases) and fucose metabolism (alpha-l-fucosidase, l-fucose isomerase) were identified in *C. acnes* isolates CBS-BPNBT19269, CBS-BPNBT19195 and CBS-BPNBT19153. Fucose utilization proteins could be involved in adherence to human skin cells and red blood cells [39], since the ability to utilize fucose has been reported to provide bacteria with a competitive advantage, contributing to heightened colonization and pathogenesis [40]. Notably, *C. acnes* isolates belonging to phylotype III are generally not associated with disease states [26], and PC isolates CBS-BPNBT19223 and CBS-BPNBT19227 notably did not carry the lysine synthesis *lysA* gene (Table 3), which has an important role in establishing bloodstream infections by *S. aureus* [41].

**Table 3.** Antimicrobial and stress resistance genes identified in *C. acnes* genomes using AMRFinderPlus

	Class	Gene	Function	Gene encoded (%)*					
				BPNBT-19269	BPNBT-19195	BPNBT-19329	BPNBT-19223	BPNBT-19227	BPNBT-19153
**Antimicrobial resistance**	Bacitracin	*bcrA*	Bacitracin resistance ABC transporter ATP-binding subunit	–	–	31.5	–	–	–
	Fosfomycin	*lmrC*	ABC-F type ribosomal protection protein	35.2	35.2	35.8	35.8	35.7	35.2
	Glycopeptide	*vanA-Ao2*	d-Alanine--(R)-lactate ligase	32.5	32.5	–	31.9	31.9	32.5
		*vanB*	d-Alanine--(R)-lactate ligase	39.5	39.8	39.8	40.1	40.1	39.8
		*vanH-D*	d-Lactate dehydrogenase	–	–	–	–	36.66	–
		*vanK-Sc*	Peptidoglycan bridge formation peptidyl transferase	52.3	52.3	52.3	52.3	52.3	52.3
		*vanR-I*	Vancomycin resistance response regulator transcription factor	39.5	39.9	40.4	37.9	37.9	39.9
		*vanR-M*	VanM-type vancomycin resistance DNA-binding response regulator	39.4	38.9	40.3	39.8	39.8	39.4
		*vanU-G*	Glycopeptide resistance transcriptional regulator	–	52.11	–	–	–	52.1
	Lincosamide/Macrolide/Streptogramin	*cipA*	23S rRNA [adenine(2503)-C(8)]-methyltransferase	–	–	34.3	34.3	34.3	–
		*cfr(D)*	23S rRNA [adenine(2503)-C(8)]-methyltransferase	34.6	34.6	–	–	–	34.6
	Macrolide	*car(A)*	ABC-F type ribosomal protection protein	32.9	32.9	32.9	–	–	32.9
		*myrA*	23S rRNA [guanine(748)-N(1)]-methyltransferase	40.0	40.0	40.6	–	–	40.0
		*ole(B)*	ABC-F type ribosomal protection protein	32.5	32.5	32.2	32.9	32.7	32.5
		*tlr(C)*	ABC-F type ribosomal protection protein	–	–	–	32.7	32.7	–
	Mupirocin	*mupA*	Mupirocin-resistant isoleucine b tRNA ligase	34.7	–	35.0	35.3	34.6	–
	Phenicol	*estDL136*	Chloramphenicol hydrolase	33.8	34.7	33.4	32.3	32.6	33.8
	Pleuromutilin	*taeA*	ABC-F type ribosomal protection protein	34.4	41.6	35.0	34.5	34.5	34.8
	Rifamycin	*helR*	RNA polymerase recycling motor ATPase	30.2	–	–	–	–	30.03
	Sulphonamide	*sul4*	Sulphonamide-resistant dihydropteroate synthase	41.6	41.6	41.6	41.3	41.6	41.6
	Tetracycline	*otr(B)*	Oxytetracycline resistance efflux MFS transporter	33.0	33.2	33.2	33.3	33.3	33.2
	Trimethoprim	*dfrA45*	Trimethoprim-resistant dihydrofolate reductase	38.0	–	–	37.4	37.4	37.4
**Stress resistance**	Arsenic	*acrB*	Arsenite efflux transporter	51.0	–	–	–	–	–
		*arsA*	Arsenite efflux transporter ATPase subunit	58.2	–	–	–	–	–
		*arsD*	Arsenite efflux transporter metallochaperone	48.0	–	–	–	–	–
	Copper	*copR*	Heavy metal response regulator transcription factor	44.4	44.9	45.3	44.9	44.9	44.9
		*cueA*	Copper resistance metal-translocating P1-type ATPase	42.1	42.2	42.5	41.5	41.5	42.2
	Copper/gold	*golT*	Gold/copper-translocating P-type ATPase	38.6	38.7	38.3	39.1	39.0	38.7
	Efflux	*smdA*	Multidrug efflux ABC transporter permease/ATP-binding subunit SmdA	31.3	31.1	31.3	30.9	30.9	30.8

*Percent identity values obtained from AMRFinder.

#### Antimicrobial resistance

Genome assessment of antimicrobial resistance elements indicates that all six PC *C. acnes* isolates harbour genetic elements associated with multidrug efflux pumps, genes associated with copper and gold resistance and resistance to a wide range of antibiotics (Table 3). These resistance mechanisms have been described to play an important role in bacterial pathogenesis, persistence and immune evasion [42,43].

Though most of the *C. acnes* isolates assessed in this study were genetically similar, isolates CBS-BPNBT19269 (phylotype IA) and CBS-BPNBT19329 (phylotype II) carry distinguishing genetic characteristics. CBS-BPNBT19269 uniquely contains a putative mobile genetic element (MGE) flanked by transposases at both 5′ and 3′ ends. This MGE encodes for genes involved in arsenite efflux and regulation (*arsA*, *arsB*, *arsD* and *asrR*) and cadmium resistance (OYC54_000139), which have been shown to contribute to persistence, resistance to benzalkonium chloride and increased virulence in other bacteria such as *Listeria monocytogenes* [39,41]. Isolate CBS-BPNBT19329 contains genes that encode for lantibiotic production genes (OYC54_00245, OYC54_002437) and the *bcrA* gene involved in bacitracin resistance. Overall, these genes have been reported to aid in environmental adaptation by pathogenic bacteria, providing pathogens with a competitive edge during host colonization and resulting in heightened virulence [44,47]. Notably, MIC data did not highlight major differences in the inhibitory concentrations against bacitracin, trimethoprim, mupirocin and rifampicin (Table 4), despite the differences observed in the presence of genetic elements that promote resistance to antibiotics (Table 3), suggesting the involvement of additional factors in the resistance against the antimicrobials tested. Importantly, the impact of potential differential expression of antimicrobial resistance genes triggered by the PC storage environment on transfusion patients remains to be studied.

**Table 4.** MIC of *C. acnes* PC isolates

Bacterial isolate	Rifampicin (µg ml^−1^)			Mupirocin (µg ml^−1^)			Trimethoprim (µg ml^−1^)			Bacitracin 10U (mm)		
	Assay 1	Assay 2	Assay 3	Assay 1	Assay 2	Assay 3	Assay 1	Assay 2	Assay 3	Assay 1	Assay 2	Assay 3
**CBS-BPNBT19269**	0.003	0.0025	0.003	>1,024	>1,024	>1,024	1.5	1.5	1.5	53	53	54
**CBS-BPNBT19195**	0.0025	0.003	0.003	>1,024	>1,024	>1,024	1.5	2	1.5	54	55	54
**CBS-BPNBT19329**	<0.002	<0.002	<0.002	>1,024	>1,024	>1,024	3	3	3	57	57	57
**CBS-BPNBT19223**	0.0025	0.003	0.0025	>1,024	>1,024	>1,024	8	8	8	60	60	62
**CBS-BPNBT19227**	<0.002	<0.002	<0.002	>1,024	>1,024	>1,024	2	2	2	62	60	59
**CBS-BPNBT19153**	0.002	0.002	0.002	>1,024	>1,024	>1,024	0.38	0.38	0.38	55	56	56
***C. acnes* ATCC 6919**	0.004	0.003	0.004	>1,024	>1,024	>1,024	0.5	0.5	0.5	48	49	49
***S. aureus* ATCC 29213**	0.008	0.008	0.004	0.09	0.125	0.125	0.5	0.5	0.5	15	14	15

### Phylogenetic comparison of PC, clinical and commensal *C. acnes* isolates

**Fig. 3.**
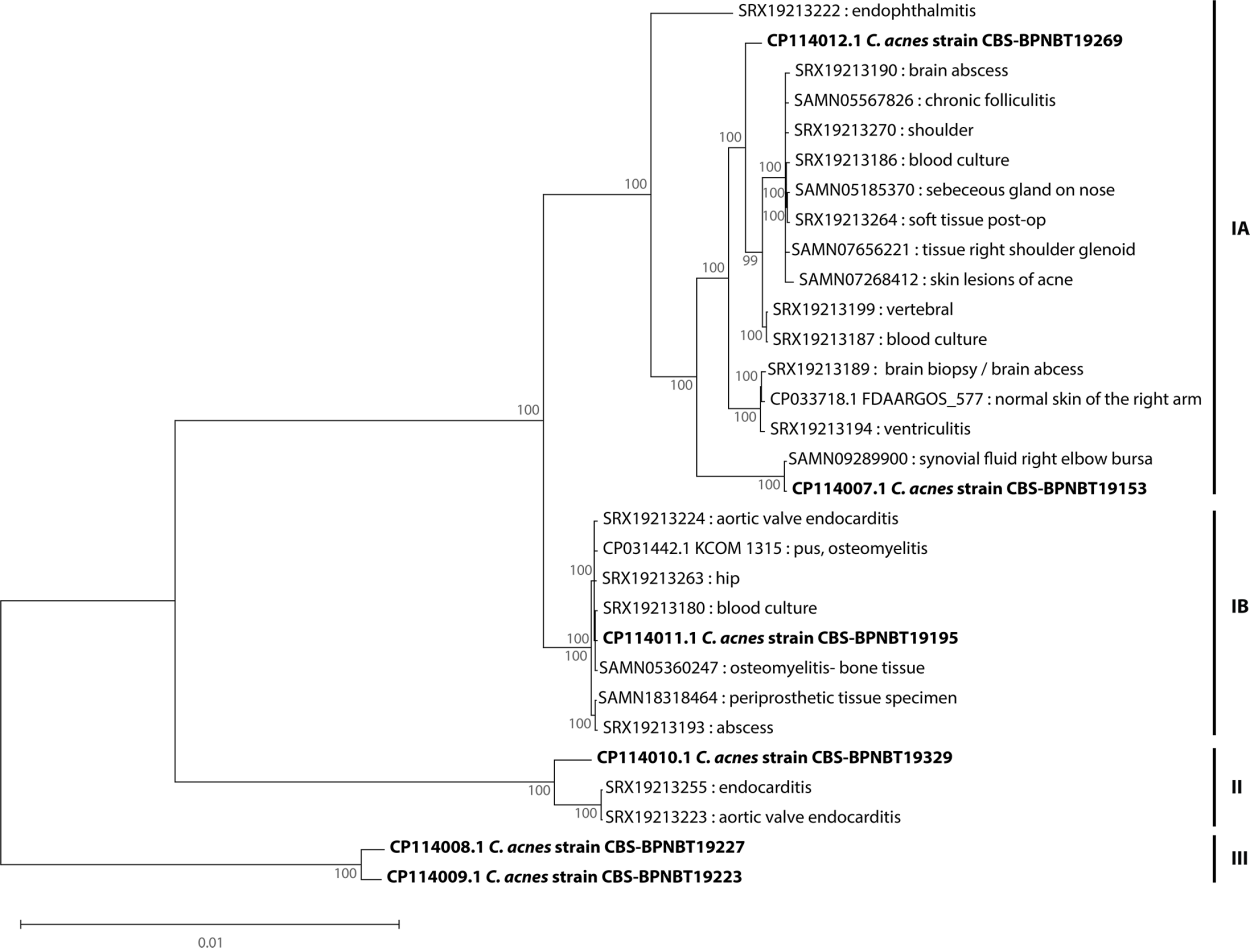
Phylogenetic analysis of *C. acnes* core genomes. The core-genome multi-alignment was generated using Parsnp and a maximum likelihood tree constructed using MEGA with 1,000 bootstrap replicates. The scale bar represents the phylogenetic distance expressed as nucleotide substitutions per site. Bootstrap values are shown at each node. Reference sequences were obtained from GenBank with tips labelled by biosample or SRA accession and host isolation site. Strains from this study are shown in bold and phylotypes for each strain are shown on the right.

A study that assessed clinical (*n*=86) and commensal isolates (*n*=103) concluded that all phylotypes except for phylotype III (*n*=8) were capable of causing infections, with phylotype IA accounting for most of the isolates tested (40%, *n*=75) [29]. A phylogenetic comparison of the PC isolates to clinical isolates involved in a wide variety of infections (Fig. 3) shows that~62.5% of the clinical samples belong to phylotype IA, which is consistent with previous reports [28]. Isolates CBS-BPNBT19269 and CBS-BPNBT19153 are closely related to clinical isolates derived from a variety of sites, including soft tissue infections and normal skin. Isolate CBS-BPNBT19195 (phylotype IB) clusters with isolates that caused deep tissue infections like osteomyelitis and endocarditis. Finally, isolate CBS-BPNBT19329 was found to be closely related to two clinical isolates from endocarditis samples. Together, these results indicate that all PC isolates that belong to phylotype I and II have the potential to cause disease. Interestingly, isolates CBS-BPNBT19227 and CBS-BPNBT19223 were genomically distinct from the clinical isolates analysed and belong to phylotype III, consistent with previous findings [29].

## Conclusions and recommendations

In this study, we isolated and sequenced six *C. acnes* genomes from contaminated PCs. Genome comparisons revealed the presence of a large invertible DNA region, and AMR and virulence analyses highlight the presence of key genes that may influence pathogenesis. Specifically, glycopeptide, macrolide, sulphonamide and tetracycline resistance genes were present in all isolates, as well as virulence factors that function in cell adherence, immune evasion, exoenzymes and secretion. Overall, this work provides in-depth genomic analysis of *C. acnes* and a more comprehensive understanding of factors that may contribute to infection by this opportunistic pathogen.

Our work has highlighted the potential clinical impact on patients receiving PC units contaminated with *C. acnes* and provides a foundation for future studies aimed at determining the impact of the PC environment on the virulence profile of *C. acnes*. Mitigation strategies are actively being developed and implemented to decrease the risk to transfusion patients. Canadian Blood Services has implemented treatment of PCs with the Pathogen Reduction Technology INTERCEPT, which has demonstrated to be effective in eliminating *C. acnes* [48]. However, for blood suppliers who have not implemented this proactive approach, the risk of transfusing *C. acnes* contaminated PCs persists. We have recently demonstrated that current skin disinfection methods are not fully effective against *C. acnes* [28], and new approaches should be considered to decrease contamination levels of blood components with this bacterium. Furthermore, we have shown that nutrient supplementation of culture media results in increased detection of *C. acnes* during PC screening [49], a method that could be adopted by blood suppliers to enhance PC safety. In conclusion, current practice during blood collection and PC testing can be improved to minimize the risk of transfusing PCs contaminated with *C. acnes*, which carry virulence factors and can potentially cause chronic infections.

## Abbreviations

AMR: antimicrobial resistance
MGE: mobile genetic element
PCs: platelet concentrates
PGAP: Prokaryotic Genome Annotation Pipeline
